# Age‐dependent electrocardiographic changes in Pgc‐1β deficient murine hearts

**DOI:** 10.1111/1440-1681.12863

**Published:** 2017-11-29

**Authors:** Shiraz Ahmad, Haseeb Valli, Samantha C Salvage, Andrew A Grace, Kamalan Jeevaratnam, Christopher L‐H Huang

**Affiliations:** ^1^ Physiological Laboratory University of Cambridge Cambridge United Kingdom; ^2^ Department of Biochemistry University of Cambridge Cambridge United Kingdom; ^3^ Faculty of Health and Medical Sciences University of Surrey Guildford Surrey United Kingdom; ^4^ PU‐RCSI School of Medicine Perdana University Selangor Darul Ehsan Malaysia

**Keywords:** cardiac arrhythmias, cardiac conduction, electrocardiogram, ECG, peroxisome proliferator activated receptor‐γ‐coactivator‐1 (PGC‐1)

## Abstract

Increasing evidence implicates chronic energetic dysfunction in human cardiac arrhythmias. Mitochondrial impairment through *Pgc‐1*β knockout is known to produce a murine arrhythmic phenotype. However, the cumulative effect of this with advancing age and its electrocardiographic basis have not been previously studied. Young (12‐16 weeks) and aged (>52 weeks), wild type (WT) (n = 5 and 8) and *Pgc‐1*β^*−/−*^ (n = 9 and 6), mice were anaesthetised and used for electrocardiographic (ECG) recordings. Time intervals separating successive ECG deflections were analysed for differences between groups before and after β1‐adrenergic (intraperitoneal dobutamine 3 mg/kg) challenge. Heart rates before dobutamine challenge were indistinguishable between groups. The *Pgc‐1*β^*−/−*^ genotype however displayed compromised nodal function in response to adrenergic challenge. This manifested as an impaired heart rate response suggesting a functional defect at the level of the sino‐atrial node, and a negative dromotropic response suggesting an atrioventricular conduction defect. Incidences of the latter were most pronounced in the aged *Pgc‐1*β^*−/−*^ mice. Moreover, *Pgc‐1*β^*−/−*^ mice displayed electrocardiographic features consistent with the existence of a pro‐arrhythmic substrate. Firstly, ventricular activation was prolonged in these mice consistent with slowed action potential conduction and is reported here for the first time. Additionally, *Pgc‐1*β^*−/−*^ mice had shorter repolarisation intervals. These were likely attributable to altered K^+^ conductance properties, ultimately resulting in a shortened QT_c_ interval, which is also known to be associated with increased arrhythmic risk. ECG analysis thus yielded electrophysiological findings bearing on potential arrhythmogenicity in intact *Pgc‐1*β^*−/−*^ systems in widespread cardiac regions.

## 1 INTRODUCTION

Cardiac arrhythmias result from disruption of the normally coordinated sequence of atrial and ventricular excitation, often following altered ion channel function. The most common of these, atrial fibrillation (AF), affects in excess of 8 million individuals in Europe, and accounts for one in three arrhythmia‐related hospital attendances. Similarly, ventricular arrhythmias are the commonest cause of sudden cardiac death (SCD), with a worldwide incidence of >300 000 deaths/year.^1^, ^2^

Previous physiological analyses of arrhythmic tendency had studied murine hearts with varying monogenic ion channel disorders modelling arrhythmic mechanisms associated with a number of inherited channelopathies. However growing evidence implicates the energetic dysfunction known to occur with both ageing and some common age‐related chronic conditions in the increased incidences of both atrial and ventricular arrhythmias with age. Chronic conditions including obesity, diabetes mellitus and heart failure themselves constitute independent pro‐arrhythmic risk factors.^3^ All these conditions are associated with deteriorating metabolic, particularly mitochondrial function. Mitochondria from cardiomyocytes of AF patients showed increased DNA damage,^4^ structural abnormalities^5^ and impaired function.^4^ Similarly, patients with inherited mitochondrial disorders such as Kearns‐Sayre Syndrome show increased risks of fatal ventricular arrhythmia.^6^

Peroxisome proliferator activated receptor‐γ coactivator‐1 (PGC‐1) transcriptional coactivators offer strategic targets for electrophysiological studies in such energetically deficient hearts. The PGC‐1 family includes PGC‐1α and PGC‐1β; these are highly expressed in oxidative tissues such as the heart, brain and skeletal muscle. They are key regulators of mitochondrial mass, function, and cellular metabolism.^7^ They interact with cardiomyocyte nuclear receptor factor‐1, estrogen related receptor‐α and peroxisome proliferator‐activated receptor‐α in increasing mitochondrial biogenesis.^8^ They also upregulate expression of nuclear and/or mitochondrial‐encoded mitochondrial proteins involved in fatty acid β‐oxidation, the tricarboxylic acid cycle and electron transport.^9^ PGC‐1 protein expression and the corresponding mitochondrial activity, is coordinated with several upstream stimuli reflecting the heart's energetic demand.^10^ Conversely, obesity, insulin resistance, type II diabetes mellitus, and ageing are associated with reduced PGC‐1 protein expression and mitochondrial dysfunction.^11^

Overexpression of some PGC‐1 family members results in increased mitochondrial density and oxidative capacity.^12^ Conversely, mice deficient in both *Pgc‐1*α and *Pgc‐1*β develop a perinatally lethal low cardiac output state and conduction disease.^13^ Cardiac phenotypes of mice lacking individual members of the PGC‐1 family are less severe. *Pgc‐1*α deficient murine hearts show normal baseline contractile function but develop cardiac failure following increased afterload.^9^ Studies on *Pgc*‐1β deficient hearts are more limited, but nevertheless report normal baseline cardiac function despite their reduced mitochondrial content, but blunted heart rate responses following adrenergic stimulation.^14^ Furthermore, Langendorff‐perfused *Pgc‐1*β^*−/−*^ hearts demonstrated increased arrhythmic propensity reflected in action potential (AP) duration alternans and increased frequencies of ventricular tachycardia (VT) following programmed electrical stimulation.^14^ Isolated *Pgc‐1*β^*−/−*^ cardiomyocytes also showed altered ion channel expression patterns, spontaneous diastolic Ca^2+^ transients, and pro‐arrhythmic after‐depolarisation events.^15^ The combination of apparently normal baseline contractile function but potentially pro‐arrhythmic electrophysiological abnormalities make *Pgc*‐1β deficient hearts attractive models to investigate roles of mitochondrial impairment in arrhythmia in an absence of a confounding cardiac failure.

Murine electrocardiography (ECG) and analysis of ECG waveforms have proven useful in investigating electrophysiological changes associated with experimental cardiac disease.^16^ Murine ECGs differ in their sinus rates and action potential waveforms^17^, ^18^ but nevertheless can be used to clarify alterations in heart rate, its variability, and timings in the cardiac excitation sequence. Thus, their P‐waves, and PR, QRS and QT intervals, continue to reflect atrial and ventricular, depolarisation and repolarisation whose alterations could reflect potentially pro‐arrhythmic electrophysiological changes. Alterations in murine ECG waveforms have thus proven useful in studying murine models for the Brugada syndrome,^19^, ^20^ long QT syndrome type 3^21^ and catecholaminergic polymorphic ventricular tachycardia.^22^

The present study investigates ECG changes associated with the energetic dysfunction occurring with *Pgc‐1*β ablation. As the underlying chronic mitochondrial lesions likely exert cumulative phenotypic effects with advancing age, we studied both young and aged, WT and genetically modified animals both at baseline and following adrenergic stress. This provided additional insights into possible interactions between genotype and ageing.

## 2 RESULTS

**Figure 1:**
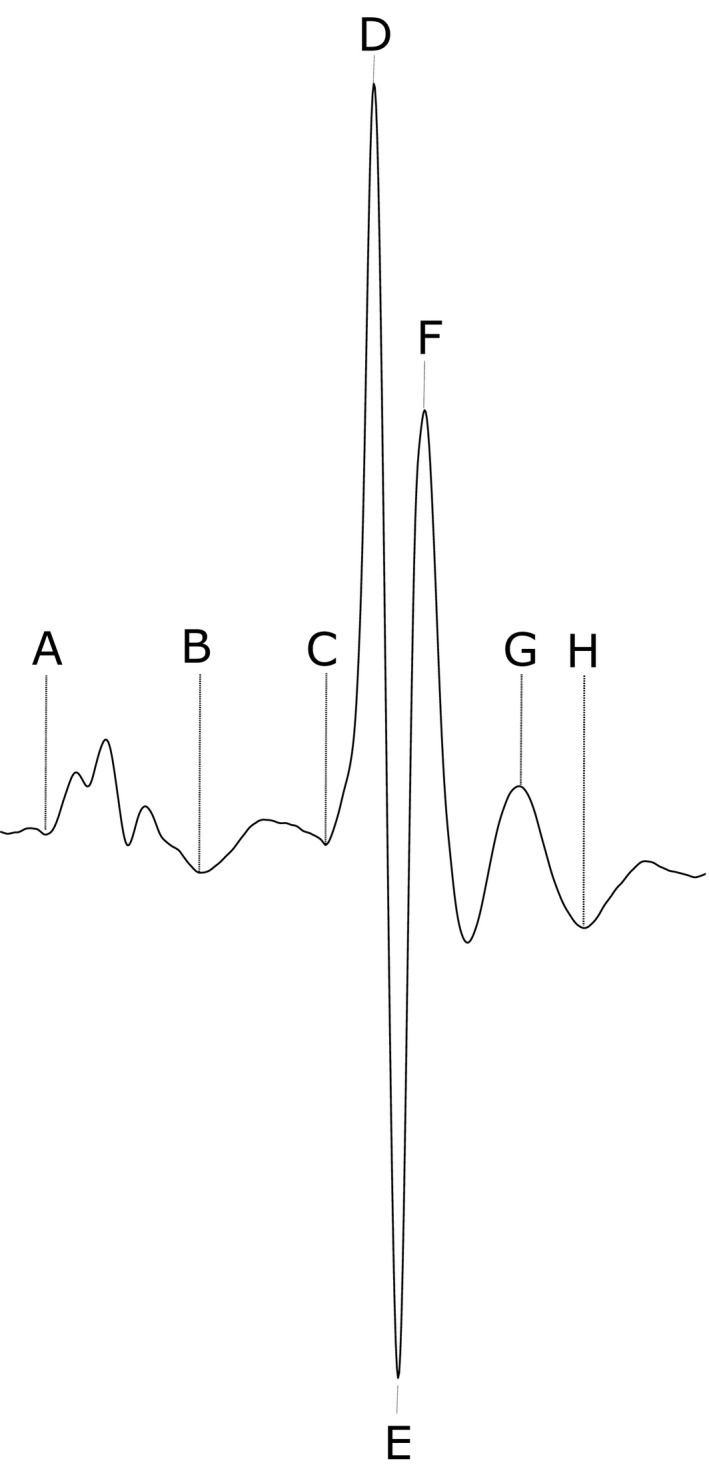
Typical ECG and definition of deflections used in quantitative analysis (A) start of P‐wave; (B) P‐wave trough/end of P‐wave; (C) start of QRS complex; (D) R wave peak; (E) trough of S wave; (F) peak of R' deflection; (G) C wave peak; (H) trough or end of C wave. The corrected QT interval, QT_c_ is taken as the interval from C to H and corrected for RR intervals^52^

**Figure 2:**
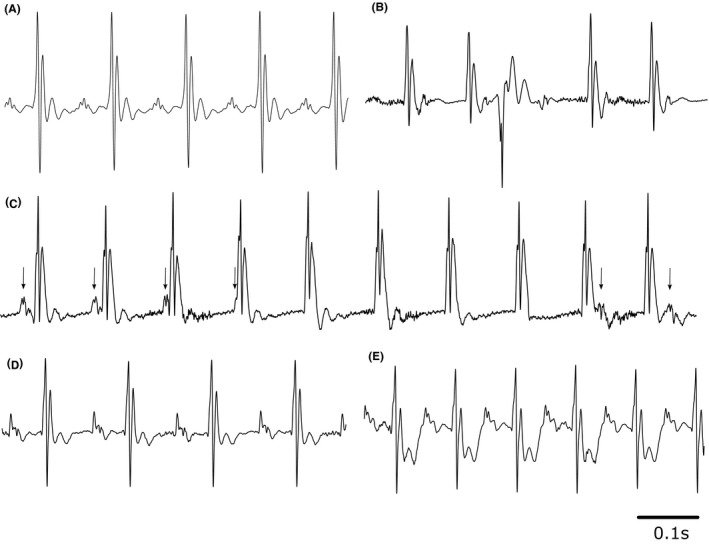
Typical ECG records from *Pgc‐1*β^*−/−*^ heart illustrating (A) normal sinus rhythm; (B) ectopic beat; (C) atrioventricular (AV) dissociation; records obtained from the same mouse. Arrows indicate timings of P‐waves (D) pre‐dobutamine and (E) following dobutamine challenge with ECG showing ST depression

There were no significant differences in weights between different experimental groups, whether stratified by age or genotype. Aged *Pgc‐1*β^*−/−*^ mice had a mean weight of 35.49 ± 1.44 g compared to 35.57 ± 1.13 g for aged WT mice. Young *Pgc‐1*β^*−/−*^ and WT mice had mean weights of 31.30 ± 1.56 g and 35.31 ± 2.26 g respectively. The complete ECG records confirmed sinus rhythm as the predominant rhythm (Figure 2A) with ECG complexes including clearly identifiable deflections as reported on earlier occasions^52^ (Figure 1). Two mice, both aged *Pgc‐1*β^*−/−*^ *,* showed multiple ectopic beats (Figure 2B) (Table 1). This initial analysis also confirmed that ST segment changes that might signal acute ischaemic change never occurred prior to dobutamine challenge. ST segment depression occurred in a small number of both WT and *Pgc‐1*β^*−/−*^ aged mice following such challenge (Figure 2D, E).

**Table 1:** Incidence of particular electrocardiographic features in the experimental groups

	WT		*Pgc‐1*β^*−/−*^	
	Young	Aged	Young	Aged
(A) Ischaemic change				
Ischaemic changes present	0	2	0	2
Ischaemic changes absent	5	6	9	4
(B) Arrhythmic ECG patterns				
Sinus rhythm only	5	4	8	3
Isorhythmic AV dissociation	0	4	1	3
Ectopic beats	0	0	0	1

Electrocardiographic records obtained at baseline prior to pharmacological intervention in young WT (n = 5), aged WT (n = 8), young *Pgc‐1*β^*−/−*^ (n = 9) and aged *Pgc‐1*β^*−/−*^ (n = 6).

### 2.1 *Pgc‐1*β^*−/−*^ hearts display impaired heart rate responses

Chronotropic incompetence is an established clinical feature of cardiac failure, although it can also occur in association with other cardiac pathology. It has been variably defined, but is commonly identified as a failure to reach an arbitrary percentage of the predicted maximum heart rate following sympathetic challenge, ranging from 70% to 85%.^23^ A previous study had reported an impaired chronotropic response in isolated ex vivo *Pgc‐1*β^*−/−*^ hearts challenged with dobutamine. These findings had not been statistically significant at a 10 ng/kg per minute infusion rate and depended on single heart rate recordings at each predefined, time point.^14^ The present experiments systematically analysed steady state parameters over 5‐minute recording periods before and following dobutamine challenge in intact animals.

**Figure 3:**
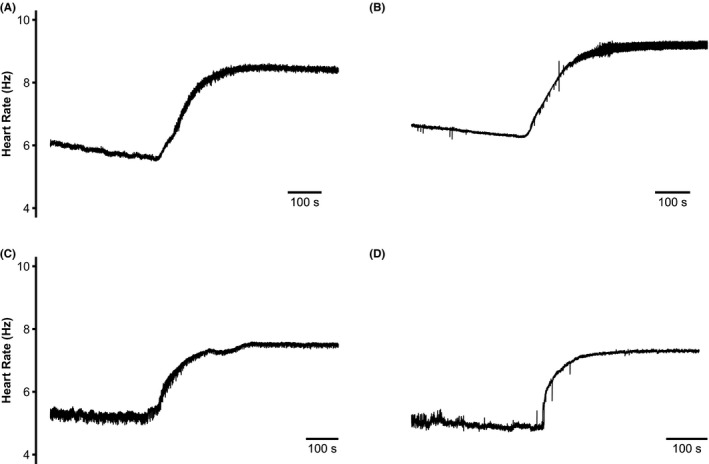
Traces plotting heart rate response curves before and following dobutamine challenge in (A) young WT, (B) aged WT; (C) young *Pgc‐1*β^*−/−*^ and (D) aged *Pgc‐1*β^*−/−*^ mouse

**Figure 4:**
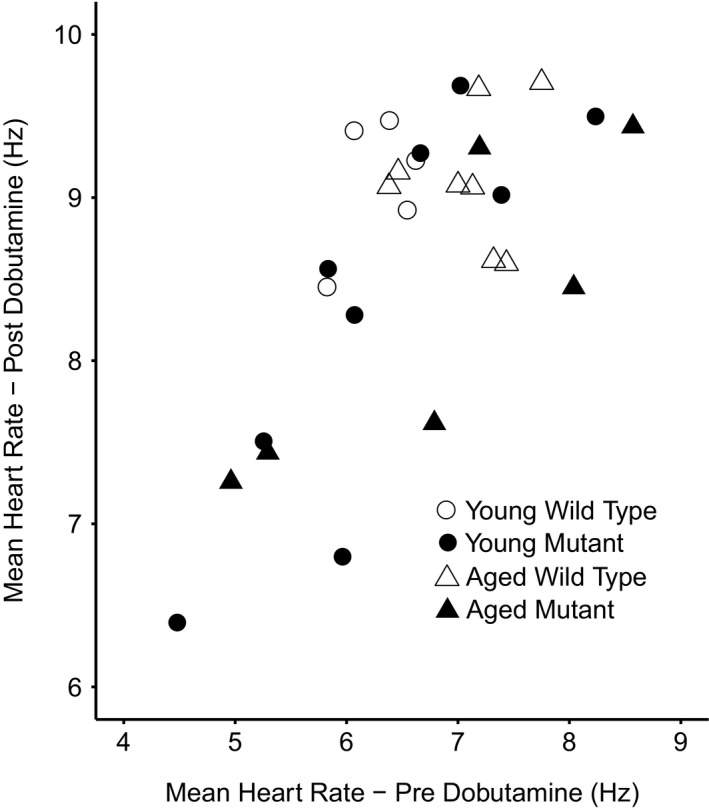
Correlations between heart rates observed pre‐ vs post‐dobutamine challenge in *Pgc‐1*β^*−/−*^ and WT

Figure 3 shows typical heart rate profiles at baseline and in response to dobutamine for each experimental group. As there is no algorithm predicting normal murine heart rates at different ages as there is with humans, we assessed the chronotropic responses to dobutamine challenge using two different parameters: (i) peak heart rate attained after dobutamine administration; and (ii) mean heart rate observed post‐dobutamine administration. Figure 4 plots mean heart rates observed before dobutamine challenge against results obtained following dobutamine challenge for each individual animal. *Pgc‐1*β^*−/−*^ animals displayed a tendency to slower basal heart rates under both conditions. A Pearson product‐moment correlation coefficient assessing the co‐variance between mean heart rates before and following dobutamine challenge demonstrated a positive association between variables, (*r* = .692, *P* < .0001). Lower resting heart rates thus correlated with lower heart rates after dobutamine challenge.

**Figure 5:**
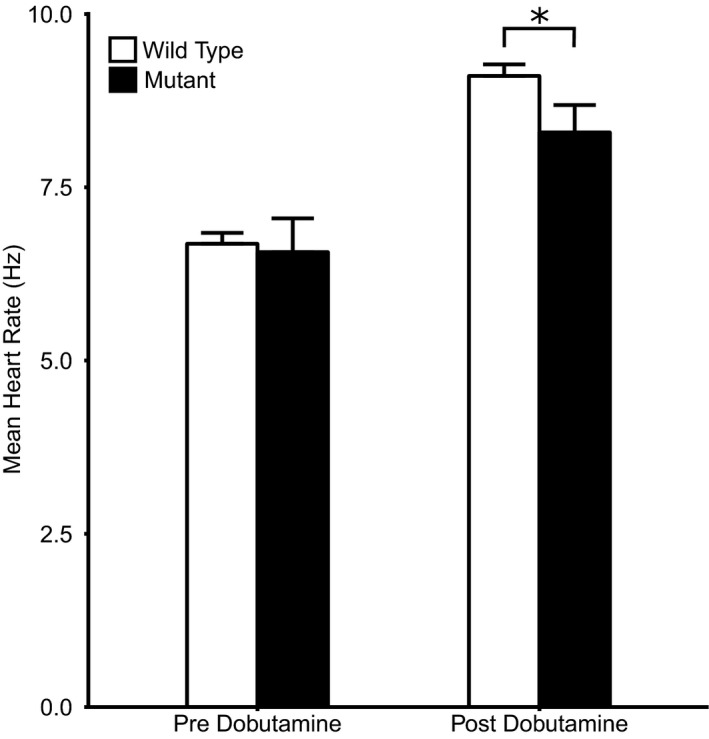
Mean heart rates in the 5 minute analysis window before and after dobutamine administration in young and aged WT and *Pgc‐1*β^*−/−*^ mice

MANOVA demonstrated significant effects of genotype (*P* = .022) and age (*P* = .048) on *steady state* heart rates (Table 2A). Post hoc Tukey tests demonstrated that genotype and age neither exerted independent nor interacting effects upon baseline steady state heart rates. In contrast, genotype exerted independent effects, with the *Pgc‐1*β^*−/−*^ mutation reducing heart rates observed following dobutamine challenge (*Pgc‐1*β^*−/−*^ 8.30 ± 0.28 Hz, n = 15; WT, 9.11 ± 0.11 Hz, n = 13; *P* = .021; Figure 5). In contrast, there were no significant effects of either age or interactions between age and genotype. Similarly, ANOVA demonstrated independent significant effects of genotype (*P* = .011) but not either age or interactive effects between genotype and age on maximum heart rates attained after dobutamine challenge. Thus, post hoc tests demonstrated lower peak heart rates in *Pgc‐1*β^*−/−*^ than WT mice (mean peak heart rate 8.47 ± 0.28 Hz vs 9.53 ± 0.21 Hz, *P* = .0084, n = 13 vs 15 respectively).

**Table 2:** Electrocardiographic features related to sino‐atrial, atrio‐ventricular and atrial conduction

	WT		*Pgc‐1*β^*−/−*^	
	Young	Aged	Young	Aged
(A) Heart rate response				
Mean heart rate prior to dobutamine challenge (Hz)	6.29 ± 0.15	7.08 ± 0.16	6.32 ± 0.38	6.81 ± 0.59
Mean heart rate following dobutamine challenge (Hz)	9.10 ± 0.19	9.12 ± 0.15	8.33 ± 0.40	8.25 ± 0.39
Peak heart rate following dobutamine challenge (Hz)	9.32 ± 0.21	9.66 ± 0.32	8.51 ± 0.40	8.41 ± 0.39
(B) Atrial conduction				
P‐wave duration prior to dobutamine challenge (ms)	26.08 ± 0.50	25.57 ± 1.06	26.06 ± 0.47	27.64 ± 0.67
P‐wave duration following dobutamine challenge (ms)	25.43 ± 0.58	26.08 ± 0.79	26.21 ± 0.48	26.90 ± 0.86
(C) AV conduction				
Mean PR interval prior to dobutamine challenge (ms)	54.20 ± 2.57	63.26 ± 4.89	56.35 ± 5.56	66.62 ± 4.25
Mean PR interval following dobutamine challenge (ms)	52.53 ± 2.22	53.61 ± 2.76	58.38 ± 5.41	76.95 ± 9.54
Hearts showing decreased PR interval following dobutamine challenge	5 of 5	6 of 6	5 of 9	1 of 6
Hearts showing increased PR interval following dobutamine challenge	0 of 5	0 of 6	4 of 9	5 of 6

Electrocardiographic features gave (A) heart rates responses in studies of young WT (n = 5), aged WT (n = 8), young *Pgc‐1*β^*−/−*^ (n = 9) and aged *Pgc‐1*β^*−/−*^ mice (n = 6), in which two of the aged WT showed AV dissociation within the ECG analysis window. Studies of atrial (B) and AV (C) conduction were therefore based on young WT (n = 5), aged WT (n = 6), young *Pgc‐1*β^*−/−*^ (n = 9) and aged *Pgc‐1*β^*−/−*^ mice (n = 6) respectively.

**Figure 6:**
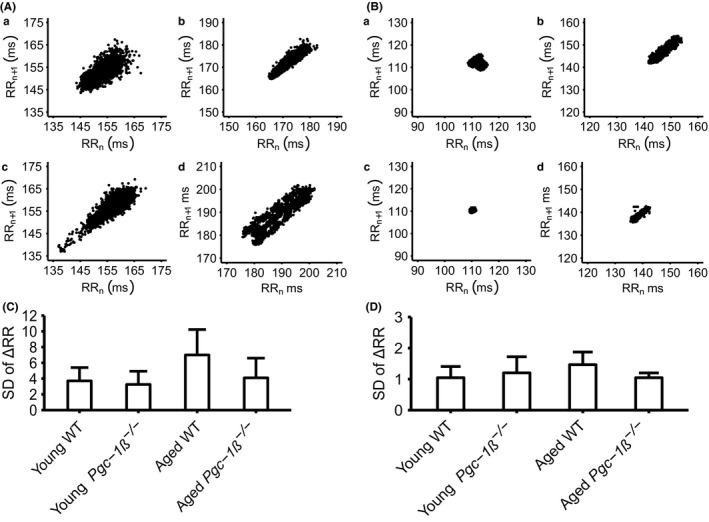
(A, B) Poincare plots pre‐ (A) and post‐dobutamine (B) in young (a,b) and aged (c,d), WT (a,c) and Pgc‐1β^‐/‐^ hearts (b,d) and (C, D) the standard deviations (SDs) of their ΔRR intervals before (C) and following dobutamine challenge (D)

Heart rate variabilities, reflecting autonomic influences on the heart, and known to be related to adverse mortality risk, expectedly altered within each experimental group following dobutamine challenge. However, the respective findings obtained before or following dobutamine did not vary between experimental groups. This was reflected in Poincare plots constructed for each mouse (Figure 6A, B), exemplified in pre‐ (A) and post‐dobutamine (B), young (a,b) and aged (c,d), WT (a,c) and *Pgc‐1*β^*−/−*^ mice (b,d). The dispersion of these points was quantified by the standard deviation of the ΔRR interval before (Figure 6C) and following dobutamine challenge (Figure 6D). ANOVA demonstrated no significant differences in such dispersions before or after dobutamine addition between different experimental groups. These results attribute the present findings to an existence of sino‐atrial node (SAN) as opposed to autonomic dysfunction in the *Pgc‐1*β^*−/−*^ mice.

### 2.2 Age‐related SA node disease in WT and *Pgc‐1*β^*−/−*^ murine hearts

Although sinus rhythm was the prevailing rhythm in all groups, both WT and *Pgc‐1*β^*−/−*^ mice demonstrated intermittent episodes of isorhythmic AV dissociation^24^ during the recording period. These episodes predominantly occurred in aged animals, affecting 3/6 aged WT mice and 4/8 aged *Pgc‐1*β^*−/−*^ mice, but in only one young *Pgc‐1*β^*−/−*^ and none of the young WT mice (Table 1). They most frequently occurred immediately following dobutamine challenge, whilst RR intervals were decreasing from their baseline, pre‐treatment values. During these episodes, RR intervals were shorter than their corresponding PP intervals, but the ventricular complexes remained identical to those observed during sinus rhythm. The latter suggests a supraventricular (likely junctional) pacemaker focus driving such activity (Figure 2C).

### 2.3 *Pgc‐1*β^*−/−*^ hearts display paradoxical atrioventricular node function

**Figure 7:**
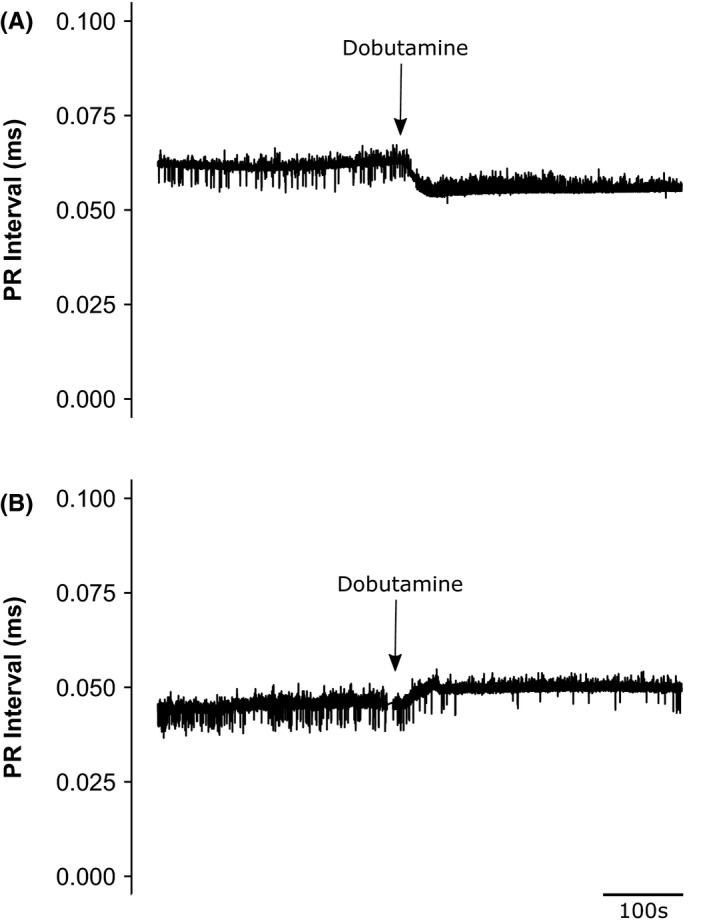
PR intervals reflecting paradoxical AV dysfunction before and following dobutamine challenge in (A) young WT and (B) aged *Pgc‐1*β^*−/−*^ mouse

MANOVA analysis indicated that P‐wave durations were not affected by either independent or interacting effects of age or genotype, whether before or following dobutamine challenge (Table 2B). In contrast, two alteration patterns of altered PR interval reflecting atrioventricular node (AVN) function were observed with dobutamine administration. These took the form of either positive or negative dromotropic effects of dobutamine appearing as increases or decreases in PR interval (Figure 7), taking place despite unchanged P‐wave durations that were similar between experimental groups suggesting a continued normal atrial conduction (Table 2B).

MANOVA indicated that neither age nor genotype affected PR interval whether before or following dobutamine challenge (Table 2C). Nevertheless, the alteration in PR interval following dobutamine challenge demonstrated differing responses from WT and *Pgc‐1*β^*−/−*^ mice. All young (5 of 5) and aged (6 of 6) WT mice showed decreased PR intervals following dobutamine challenge. In contrast, the PR interval decreased in 5 of 9 and increased in the remaining four young *Pgc‐1*β^*−/−*^ mice. In aged *Pgc‐1*β^*−/−*^ mice the result was even more marked with only one mouse showing the expected positive dromotropic effect with dobutamine administration and 5 of 6 mice showing a paradoxical negative dromotropic effect in response to dobutamine. In addition to the SAN dysfunction seen with the *Pgc‐1*β knockout there was compromised AVN conduction in a subset of mutant hearts, an effect exacerbated by increasing age.

The presence of AVN dysfunction in mutant mice which also demonstrated impaired heart rate responses led us to examine whether paradoxical AV node dysfunction underlies or is associated with the blunted chronotropic responses. Such a comparison demonstrated that *Pgc‐1*β^*−/−*^ animals with a normal AVN response showed a mean heart rate of 9.10 ± 0.22 Hz (n = 6) following dobutamine challenge. In contrast, *Pgc‐1*β^*−/−*^ animals with a paradoxical AVN response to dobutamine showed a heart rate of 7.77 ± 0.34 Hz (n = 9). A two‐tailed student *t* test confirmed that the difference was significant (*P* = .0061). Thus these findings suggest that the *Pgc‐1*β^*−/−*^ mutation is associated only with significantly altered AV nodal function in a subset of mutant mice, and that the presence of AV nodal dysfunction itself may be a marker for impaired heart rate responses.

### 2.4 Aged *Pgc‐1*β^*−/−*^ hearts display slowed ventricular activation

Ventricular activation is a synchronised, sequential process, whose onset is easily detected as the beginning of the Q wave deflection (Figure 1). Ventricular recovery is considered to begin at a timepoint between the S wave trough, and the beginning of the R' peak. Examination of three independent ECG indicators, of QR, QS and QR' durations, of ventricular activation, suggested interacting effects of age and genotype upon its duration (Table 3). MANOVA demonstrated that genotype and age, whilst not exerting independent effects, showed interacting effects upon all the three parameters of QR (*P* = .032), QS (*P* = .040) and QR' duration (*P* = .039). This was also the case with QR, QS, but not QR' (QR: *P* = .029; QS: *P* = .029; QR': *P* = .086) prior to and with all three parameters following (QR: *P* = .016; QS: *P* = .022; QR': *P* = .026) dobutamine challenge. Post hoc Tukey tests then demonstrated that prior to dobutamine administration, QR durations were longer in aged *Pgc‐1*β^*−/−*^ than aged WT (*P* = .030), with indications of such differences in aged compared to young *Pgc‐1*β^*−/−*^ mice (*P* = .059). QS intervals were longer in aged *Pgc‐1*β^*−/−*^ than either young *Pgc‐1*β^*−/−*^ (*P* = .040) or aged WT mice (*P* = .024). Following dobutamine administration, QR durations were longer in aged *Pgc‐1*β^*−/−*^ than either aged WT (*P* = .035) or young *Pgc‐1*β^*−/−*^ (*P* = .030). QS durations were longer in aged *Pgc‐1*β^*−/−*^ than either young *Pgc‐1*β^*−/−*^ (*P* = .035) or aged WT mice (*P* = .026). QR' durations were longer in aged *Pgc‐1*β^*−/−*^ mice than either aged WT (*P* = .039) or young *Pgc‐1*β^*−/−*^ mice (*P* = .017).

**Table 3:** Electrocardiographic activation intervals

	WT		*Pgc‐1*β^*−/−*^	
	Young	Aged	Young	Aged
QR duration before dobutamine challenge (ms)	6.85 ± 0.67	5.89 ± 0.63	6.20 ± 0.48	8.35 ± 0.52
QR duration following dobutamine challenge (ms)	7.14 ± 0.75	6.12 ± 0.60	6.12 ± 0.48	8.56 ± 0.56
QS duration before dobutamine challenge (ms)	10.19 ± 0.47	9.43 ± 0.45	9.67 ± 0.45	11.78 ± 0.7
QS duration following dobutamine challenge (ms)	10.60 ± 0.62	9.76 ± 0.40	9.91 ± 0.43	12.07 ± 0.77
QR' duration before dobutamine challenge (ms)	14.24 ± 0.60	14.22 ± 0.40	13.82 ± 0.34	16.20 ± 0.92
QR' duration following dobutamine challenge (ms)	14.95 ± 0.41	14.39 ± 0.55	14.15 ± 0.38	16.72 ± 0.89

Electrocardiographic measurements made in QR, QS and QR' durations before and following dobutamine challenge in young WT (n = 5), aged WT (n = 8), young *Pgc‐1*β^*−*/*−*^ (n = 9) and aged *Pgc‐1*β^*−*/*−*^ mice (n = 6).

### 2.5 *Pgc‐1*β^*−/−*^ hearts show shortened ventricular recovery times after adrenergic challenge

Age and genotype exerted contrasting effects on ventricular recovery times. Genotype affected all three measures of such recovery (RT_c_, R'T_c_ and ST_c_ durations; *P* = .0098, *P* = .0014 and *P* = .0029, respectively) (Table 4). In contrast, age did not significantly affect any of these recovery parameters, nor were there any interactive effects of age and genotype. Post hoc testing showed that (for all parameters) the difference lay in findings obtained post‐dobutamine challenge; there were no differences due to age, genotype or their interaction prior to dobutamine administration. Following dobutamine administration all three parameters showed a marked effect of genotype (*P* = .015, *P* = .021 and *P* = .0067 respectively) but no other effects. Post hoc Tukey tests showed that *Pgc‐1*β^*−/−*^ showed significantly shorter RT_c_, R'T_c_ and ST_c_ intervals than WT mice (*P* = .0053, *P* = .018 and *P* = .0080 respectively). Thus, all three recovery parameters were highly concordant confirming that the *Pgc‐1*β ablation significantly shortened ventricular recovery parameters (Table 5).

**Table 4:** Electrocardiographic recovery intervals

	WT		*Pgc‐1*β^*−/−*^	
	Young	Aged	Young	Aged
RT_c_ duration before dobutamine challenge (ms)	29.00 ± 0.54	30.60 ± 0.87	28.51 ± 1.05	28.89 ± 0.87
RT_c_ duration following dobutamine challenge (ms)	33.43 ± 0.77	33.75 ± 0.44	31.41 ± 0.86	31.79 ± 0.41
R'T_c_ duration before dobutamine challenge (ms)	23.15 ± 0.45	23.64 ± 0.71	22.55 ± 0.91	22.55 ± 0.56
R'T_c_ duration following dobutamine challenge (ms)	25.97 ± 0.43	25.88 ± 0.55	24.16 ± 0.66	24.40 ± 0.29
ST_c_ duration before dobutamine challenge (ms)	26.35 ± 0.38	27.65 ± 0.77	25.79 ± 0.92	26.06 ± 0.55
ST_c_ duration following dobutamine challenge (ms)	30.13 ± 0.68	30.31 ± 0.50	28.00 ± 0.71	28.40 ± 0.30

Electrocardiographic measurements made in RT_c_, R'T_c_ and ST_c_ durations before and following dobutamine challenge in young WT (n = 5), aged WT (n = 8), young *Pgc‐1*β^*−*/*−*^ (n = 9) and aged *Pgc‐1*β^*−*/*−*^ (n = 6) mice. One young and one aged *Pgc‐1*β^*−*/*−*^ mouse were excluded as these showed paradoxical dromotropic effects that lead to prolonged PR intervals and P waves that interfered with determinations of the end of the C wave to give the following n values: young WT (n = 5), aged WT (n = 8), young *Pgc‐1*β^*−*/*−*^ (n = 8) and aged *Pgc‐1*β^*−*/*−*^ (n = 5).

**Table 5:** Electrocardiographic recovery intervals: WT and *Pgc1*β^*−/−*^ compared

	WT	*Pgc‐1*β^*−/−*^
RT_c_ duration before dobutamine challenge (ms)	29.99 ± 0.60	28.65 ± 0.70
RT_c_ duration following dobutamine challenge (ms)	33.63 ± 0.38	31.53 ± 0.57
R'T_c_ duration before dobutamine challenge (ms)	23.45 ± 0.46	22.55 ± 0.58
R'T_c_ duration following dobutamine challenge (ms)	25.91 ± 0.37	24.24 ± 0.44
ST_c_ duration before dobutamine challenge (ms)	27.15 ± 0.51	25.89 ± 0.59
ST_c_ duration following dobutamine challenge (ms)	30.24 ± 0.39	28.13 ± 0.48

Electrocardiographic measurements made in RT_c_, R'T_c_ and ST_c_ durations before and following dobutamine challenge in young WT (n = 5), aged WT (n = 8), young *Pgc‐1*β^*−*/*−*^ (n = 9) and aged *Pgc‐1*β^*−*/*−*^ mouse (n = 6). One young and one aged *Pgc‐1*β^*−*/*−*^ mouse were excluded as these showed paradoxical dromotropic effects that led to prolonged PR intervals and P waves that interfered with determinations of the end of the C wave to give the following n values: young WT (n = 5), aged WT (n = 8), young *Pgc‐1*β^*−*/*−*^ (n = 8) and aged *Pgc‐1*β^*−*/*−*^ (n = 5). This gave total n values of WT and *Pgc‐1*β^*−*/*−*^ of 13 in both cases.

### 2.6 Emergence of a short‐QT phenotype in *Pgc‐1*β^*−/−*^ animals

The QT_c_ interval has traditionally been used as a marker for repolarisation abnormalities in that the electrocardiographic phenotype is usually caused by a defect in ventricular recovery. However, it is more accurate to describe the QT_c_ interval as a parameter that describes the combined durations of both activation and recovery *i.e*. the duration of ventricular excitation. The onset of ventricular activation is represented by the Q wave deflection; the C wave trough was taken to represent the end of ventricular recovery and hence used for calculation of the QT interval in the present study (Figure 1). Genotype, but neither age (*P *= .083) nor interactions between age and genotype (*P* = .075), significantly affected QT_c_ interval (*P* = .0071) (Table 6). ANOVA indicated no differences between groups at baseline. Following dobutamine challenge, genotype (*P* = .032), but not age affected QT_c_ interval. Post hoc Tukey HSD tests revealed that, following dobutamine administration, young *Pgc‐1*β^*−/−*^ had shorter QT_c_ intervals than both young WT (*P* = .026) and aged WT mice (*P* = .041). There was also a trend towards young *Pgc‐1*β^*−/−*^ mice having shorter QT_c_ intervals than aged *Pgc‐1*β^*−/−*^ mice (*P* = .094). Thus, *Pgc‐1*β^*−/−*^ mice had shorter QT_c_ intervals than their WT counterparts with most of the effect arising from shortening of the QT_c_ intervals in young *Pgc‐1*β^*−/−*^ mice.

**Table 6:** Mean electrocardiographic QT_c_ durations

	WT		*Pgc‐1*β^*−/−*^	
	Young	Aged	Young	Aged
Mean QT_c_ before dobutamine challenge (ms)	34.43 ± 0.20	35.60 ± 0.79	33.37 ± 1.12	35.78 ± 1.18
Mean QT_c_ following dobutamine challenge (ms)	40.23 ± 0.45	39.64 ± 0.52	37.01 ± 0.87	39.77 ± 0.81

Electrocardiographic measurements made in QT_c_ durations before and following dobutamine challenge in young WT (n = 5), aged WT (n = 8), young *Pgc‐1*β^*−*/*−*^ (n = 9) and aged *Pgc‐1*β^*−*/*−*^ (n = 6) mice. One young and one aged *Pgc‐1*β^*−*/*−*^ mouse were excluded as these showed paradoxical dromotropic effects that led to prolonged PR intervals and P waves that interfered with determinations of the end of the C wave to give the following n values: young WT (n = 5), aged WT (n = 8), young *Pgc‐1*β^*−*/*−*^ (n = 8) and aged *Pgc‐1*β^*−*/*−*^ (n = 5). This gave total n values of WT and *Pgc‐1*β^*−*/*−*^ of 13 in both cases.

## 3 DISCUSSION

The ECG yields much prescient and strategic clinical electrophysiological information as a primary investigational tool. Its recent experimental application had demonstrated valuable insights into electrophysiological abnormalities in murine hearts modelling clinical arrhythmic conditions. Cellular energetic dysfunction following metabolic disturbances is increasingly recognised as important factor in the aetiology of such atrial and ventricular arrhythmias. Destabilisation of inner mitochondrial membrane potentials results in approximately 10‐fold increases in reactive oxygen species production,^25^ in turn affecting maximum Na^+^ 25 and K^+^ current,^26^ sarcolemmal K_ATP_ channel function, Na^+^ and L‐type Ca^2+^ channel inactivation kinetics and late Na^+^ current. They also affect ryanodine receptor function alterations in which affect surface membrane excitability and intracellular Ca^2+^ homeostasis.^27^, ^28^, ^29^ Mitochondria are also the main cardiomyocyte ATP source and ATP/ADP depletion increases sarcolemmal ATP‐sensitive K^+^ channel (sarcK_ATP_) open probabilities affecting action potential duration (APD), effective refractory period (ERP) and heterogenous current sinks potentially causing current‐load mismatch.^30^ These cellular mechanisms could in turn potentially give rise to potentially pro‐arrhythmic effects on cell‐cell coupling,^31^ AP conduction^25^ and AP repolarisation.^26^ There may also be an appearance of alternans and Ca^2+^ mediated triggering phenomena.^27^ The *Pgc‐1*β genetic modification has thus been associated with altered ion channel function and ventricular arrhythmias in Langendorff‐perfused heart preparations.^15^

ECG alterations accompanying the associated mitochondrial dysfunction were therefore investigated in intact anaesthetised *Pgc‐1*β^*−/−*^ mice lacking the transcriptional coactivator *Pgc‐1*β. The present study therefore yielded electrophysiological features associated with *Pgc‐1*β ablation in the in vivo system with intact autonomic innervation and normal cardiac mechanical function, building upon earlier reports from the cellular studies^15^ and ex vivo hearts.^14^ The pharmacological manoeuvres involving dobutamine challenge in the latter studies would largely involve β1‐adrenergic receptor activity, whereas the present in vivo studies could potentially further involve β2‐adrenergic receptor mediated extracardiac changes which in the clinical setting are also known to influence arrhythmic risk.

The present experiments characterised the intervals separating specific ECG waveform components more closely than did previous studies. Quantitative statistical analysis of these steady state parameters then employed two way factorial MANOVA testing for interacting and non‐interacting effects of age and genotype before and after dobutamine challenge. The presence of significant differences then prompted further, two way factorial ANOVA to ascertain whether the difference occurred before or following dobutamine application. Finally, appropriate Tukey HSD tests assessed for particular differences between individual parameters. Peak heart rates following dobutamine challenge were analysed by themselves by a two way factorial ANOVA followed by post hoc Tukey tests.

The ECG analysis demonstrated a range of age‐dependent abnormalities associated with the *Pgc‐1*β^*−/−*^ genotype. The predominant ECG pattern in both young and aged, WT and *Pgc‐1*β^*−/−*^ mice was one of sinus rhythm. Any ischaemic ECG changes observed were associated with age but were not specific to *Pgc‐1*β^*−/−*^ or WT genotypes, suggesting that there was no underlying difference in vascular as opposed to primary cardiomyocyte function between the two groups. However, we demonstrated for the first time blunted chronotropic responses to dobutamine challenge in an intact *Pgc‐1*β^*−/−*^ mammalian system despite heart rate variabilities suggesting unchanged autonomic backgrounds. Previous reports had demonstrated compromised heart rate responses in ex vivo Langendorff‐perfused *Pgc‐1*β^*−/−*^ hearts following dobutamine challenge.^14^ These results together suggest that the impaired heart rate response of *Pgc‐1*β^*−/−*^ hearts does not reflect generalised autonomic dysfunction but rather alterations in the intrinsic myocardial response to dobutamine. Ageing did not affect this chronotropic response, implicating the mutation as opposed to background deterioration of maximal heart rate with age.

Aged mice, independent of genotype, also showed episodes of isorhythmic AV dissociation with dobutamine challenge. During these episodes regular ventricular responses were seen, with normal, narrow QRS complexes despite the absence of a fixed PR interval, even when P‐wave complexes were buried within the ventricular signal. These findings suggest that the murine SAN is vulnerable to degenerative changes with age, with appearances of supraventricular, most likely junctional, pacemaker foci intermittently dictating the ventricular rate. *Pgc‐1*β^*−/−*^ ablation also appeared to cause a more generalised defect also affecting the AVN. A significant proportion of *Pgc‐1*β^*−/−*^, particularly aged, mutants, demonstrated an abnormal negatively dromotropic response to dobutamine challenge, suggesting progressive deterioration in AVN function in *Pgc‐1*β^*−/−*^ hearts with age. Furthermore, mice showing this abnormal AVN function showed more pronounced blunting in their chronotropic response to dobutamine, relating an AVN dysfunction to the chronotropic deficit observed here.

Pathological bradycardic rhythms secondary to cardiac conduction system disease are known to occur with ageing, often necessitating permanent pacemaker implantation. Progressive fibrotic change is a recognised feature of cardiac ageing in both animal^32^, ^33^ and human studies.^34^ Fibrotic change could directly disrupt gap junction function, increasing tissue resistance,^35^ or increase fibroblast‐cardiomyocyte coupling, increasing effective membrane capacitance.^36^ More recent studies indeed implicate abnormal gap junction function in both SAN and AVN disease.^37^, ^38^ Interestingly, oxidative stress, associated with mitochondrial impairment increases transforming growth factor (TGF)‐β activity,^39^ in turn implicated in such age‐related myocardial fibrosis.^40^ Conversely augmented mitochondrial anti‐oxidant capacity protects against features of cardiac ageing including fibrotic change.^41^ Mitochondrial dysfunction could also impair gap junction function through elevating intracellular [Ca^2+^] or altering connexin phosphorylation through oxidative stress.^42^ Finally, the range of ionic currents including RyR2 channel function^43^ involved in SAN and AVN activity, are potentially modifiable by mitochondrial dysfunction. Isolated cardiomyocytes from *Pgc‐1*β^*−/−*^ hearts have previously been reported to display altered diastolic Ca^2+^ transients in keeping with abnormal *RyR2* function.^15^

ECG deflections related to ventricular activation and recovery confirmed previous reports that murine ECGs lack well‐defined ST segments.^17^, ^44^ The murine ECG shows a R' wave deflection, immediately following the S wave not seen in the human ECG. This is followed by a further but variably reported deflection which has not been systematically identified or formally correlated with particular action potential components. This variability may be attributed to the greater rostro‐caudal anatomical alignment of the mouse heart in the thoracic cavity and variations in limb positioning during experimental recording between reports, with consequent variations in the effective positioning of the centre of the Einthoven triangle relative to the heart. Thus, although we also identified C waves, small changes in lead positioning could lead to its apparent disappearance in one or both ECG leads. This may account for the controversy concerning its inconsistent appearance.^17^

The onset of ventricular recovery in the murine ECG has been considered to occur from time points ranging from the S wave nadir to the R' peak. A number of authors have suggested that the late component of the R' wave or the R' wave in totality is in fact part of ventricular repolarisation.^16^, ^18^, ^44^ Others suggested that inclusion of the R' wave may lead to systematic overestimation of ventricular activation, whilst its exclusion in genetically modified mouse models, such as that of the Brugada syndrome, which displays slowed conduction, may lead to underestimation of ventricular activation times.^18^

We accordingly explored a set of related recovery parameters that utilised both the S wave nadir and peak of the R' wave as cut‐off separating ventricular activation and recovery phases. Each parameter is likely to capture activation and recovery in different areas of myocardium, reflecting the non‐simultaneous nature of electrical activity in the myocardium. This increased the robustness of our analysis and permitted us to assess the possibility of early repolarisation in our genetic model. The statistical analysis of the different parameters of recovery and activation were highly concordant. *Pgc‐1*β^*−/−*^ hearts showed a prolongation of all such measures of ventricular activation with age whether before or following adrenergic stress, in an absence of independent effects of age or genotype. These findings parallel the reduced conduction velocities reported in other arrhythmic genetic models. These had accompanied either fibrotic change or reduced Na^+^ currents resulting from Nav1.5 deficiency in *Scn5a* ^+/*−*^ 32, 33, 45 and *Scn5a* ^+/ΔKPQ^,^21^ or secondary to Ca^2+^ handling abnormalities in *RyR2‐P2328S* hearts.^44^, ^46^, ^47^ These findings are also compatible with reports that mitochondrial abnormalities could alter *I*_K_ and therefore result in current‐load mismatch.^6^, ^30^

*Pgc‐1*β^*−/−*^ hearts also showed shorter recovery parameters than WT after dobutamine administration, without effects of age whether acting independently or interacting with genotype. Such shortened repolarisation intervals have also been implicated in arrhythmic risk. Human short QT syndrome is diagnosed using the J point to T peak interval that may represent the interval between the end of the ventricular complex to the peak of the repolarisation wave.^48^ Short QT syndrome has been traced to HERG and other K^+^ channel mutations and more recently, Ca^2+^ channel function.^49^ The present findings are thus consistent with reported alterations in K^+^ conductance properties in the *Pgc‐1*β^*−/−*^ system that would also modify current‐load matching.^15^ These changes appeared to result in shortened QT_c_ intervals for mutant mice with adrenergic stress. Although the mechanisms underlying these changes remain unclear, increased expression of *Kcna5* was reported in the latter study and may contribute to the increased K^+^ conductance observed. Additionally, the opening and K^+^ conductance of the sarcK_ATP_ is linked to rising cellular ADP levels, therefore correlating its activity to cellular metabolic status. Its activity is known to reduce the action potential duration and is thought to contribute to increased arrhythmic risk.^50^ Oxidative stress is also known to enhance sarcK_ATP_ activity; however the cellular mechanism are yet to be established but may occur through depletion of cellular ATP. Nevertheless, the effects of ROS upon sarcK_ATP_ activity could be attenuated through inhibition of protein kinase C, protein kinase G and calcium‐calmodulin kinase II but not protein kinase A, providing some insights into the pathways involved.^51^

Finally, these findings prompted us to measure QT_c_ intervals reflecting the total activation times of the ventricular myocardium. *Pgc‐1*β^*−/−*^ mice showed shorter QT_c_ intervals than their WT counterparts. Most of this effect seemed to arise in the young mutant mice, though this was not significant. This is in contrast to the shortened repolarisation parameters in both young and aged *Pgc‐1*β^*−/−*^. This likely reflects the additional, prolonged, depolarisation parameters in aged *Pgc‐1*β^*−/−*^ mice, offsetting to some degree the shortening in the repolarisation parameters.

In summary, ECG analysis demonstrates a range of electrocardiographic abnormalities associated with the *Pgc‐1*β^*−/−*^ genotype and those features particularly vulnerable to advanced age. Thus, *Pgc‐1*β^*−/−*^ mice show reduced sino‐atrial responses to dobutamine, paradoxical atrioventricular nodal function increasing in prevalence with age, slowed ventricular activation with ageing and shortened recovery parameters after dobutamine challenge.

## 4 METHODS

### 4.1 Animals

This research has been regulated under the Animals (Scientific Procedures) Act 1986 Amendment Regulations 2012 following ethical review by the University of Cambridge Animal Welfare and Ethical Review Body (AWERB). The experiments also conformed to the Guide for the Care and Use of Laboratory Animals, US National Institutes of Health (NIH Publication No. 85‐23, revised 1996). Mice from a consistent C57/B6 background to avoid possible strain‐related confounds, were housed in an animal facility at 21°C with 12‐hour light/dark cycles. Animals were fed sterile chow (RM3 Maintenance Diet, SDS, Witham, Essex, UK) and had free access to water. Wild Type C57/B6 and *Pgc‐1*β^*−/−*^ adult mice were bred for the experimental protocols. Mice were divided into four groups: groups 1 and 2 consisted of mice aged between 12 and 16 weeks, and consisted respectively of littermate WT controls (n = 5) and *Pgc‐1*β^*−/−*^ mice (n = 9). Group 3 was composed of aged (greater than 52 weeks) littermate WT controls (n = 8). Group 4 consisted of *Pgc‐1*β^*−/−*^ mice of age similarly greater than 52 weeks (n = 6). These timings parallel those used in previous studies in transgenic *Scn5a* ^+/*−*^ mice which reported increased fibrotic and electrophysiological alterations at ages above 12 months.^45^ The latter two groups used here accordingly consisted of animals above this age to investigate the effects of age on electrocardiographic parameters in these mice.

### 4.2 Electrocardiography

Mice were anaesthetised with tribromoethanol (avertin: 2,2,2 trimethylethanol, Sigma Aldrich, Poole, UK) administered into the intraperitoneal space with a 27G hypodermic needle. They were then weighed, placed supine on a warmed (37°C) platform, and their limbs secured with adhesive tape to minimise movement artefact and enable correct lead positioning. Three 2‐millimetre diameter electrodes (MLA1204; ADInstruments, Colorado Springs, CO, USA) placed in the right forelimb, left forelimb and left hindlimb respectively enabled lead I and lead II ECG recordings. The electrodes were connected to a 4‐channel NL844 pre‐amplifier whose outputs were then led through 4‐channel NL820 isolator and NL135 low‐pass filter units (set at a 1.0‐kHz cut‐off and with a 50‐Hz notch) within a NL900D chassis and power supply (Neurolog‐Digitimer, Hertfordshire, UK). To reduce electrical noise, all recordings were carried out within a grounded Faraday cage. Signals were sampled at 5 kHz and analogue‐to‐digital conversion employed a CED 1401c interface (Cambridge Electronic Design, Cambridge, UK). This then conveyed the signal to a computer for display and recording using Spike II software (Cambridge Electronic Design).

ECG recordings commenced immediately following electrode attachment in anaesthetised animals. These were continued for 5 minutes to permit preparations to reach a steady state, and for a further 5 minutes to obtain traces for quantitative analysis. Dobutamine hydrochloride (3 mg/kg; Sigma Aldrich) was then administered into the intraperitoneal space**.** ECG recording was then continued until a new steady state was reached. A further 5 minute recording period provided traces for quantitative ECG analysis following pharmacological challenge.

### 4.3 Digital signal processing

All analysis employed custom‐written software in the open‐source R programming language. Data was imported into the R program. An infinite impulse response (IIR) high pass Butterworth filter of order 2 was designed and the signal passed through to eliminate baseline drift. The signal was filtered in both forward and reverse directions to negate effects of any phase shift. A low‐pass Savitzky‐Golay algorithm was then applied. R peak fiducial points were identified with a peak‐finding algorithm used to detect QRS complexes. An iterative process determined the signal envelope and an adaptive threshold was used to identify time points in the signal that were greater than threshold in the continuous ECG signal. QRS complex timing positions were determined; peaks on a more substantive upward or downward trend were disregarded in favour of the peak with the larger absolute value. If multiple peaks were detected within 2 milliseconds the peaks were analysed again and the larger maximum value taken as the correct peak, and the smaller peak discarded. In addition, all detections were visually verified. P‐waves were analysed independently of QRS complexes by deleting the QRS complexes and subsequent isolation of the P‐wave parameters. This analysis thus made no assumption of QRS complexes being preceded by P‐waves. Analysis was performed on 300 second periods of ECG data immediately prior to and following administration of dobutamine hydrochloride. The effect of dobutamine was judged from observed ECG parameter changes. Figure 1 depicts a typical murine ECG recording with parameters calculated based upon this archetypal signal. Intervals were corrected using the formula previously described.^52^

### 4.4 Statistical analysis

Statistical analysis used the R programming language. Data sets were first tested for normality with the Shapiro‐Wilk test before statistical analysis using two way factorial multivariate analysis of variance, i.e. MANOVA with Pillai trace. The data sets analysed were the steady state heart rates, P‐wave durations, PR intervals, activation parameters of QR, QS and QR' durations, recovery parameters of RT_c_, R'T_c_ and ST_c_ durations, as well as the QT_c_ interval. Each of these were measured from ECG records of young and aged, WT and *Pgc‐1*β^*−/−*^ mice respectively, both before and following dobutamine challenge. The initial MANOVA tests examined each parameter for significant effects of age, genotype or interactive effects of age and genotype either prior to or following dobutamine challenge. Where MANOVA testing indicated existence of significant differences prior to dobutamine administration, further ANOVA analyses were conducted on pre‐drug parameters testing for effects of genotype, age or interacting effects of the two. The presence of significant effects then prompted pairwise Tukey honest significant difference testing of differences between pairs of individual parameters. Similarly, where significant differences were indicated post‐dobutamine, a similar procedure of significance testing was performed examining for significant effects post drug challenge. Peak heart rates that were obtained following dobutamine challenge, were analysed by a two way factorial ANOVA: there was no meaningful peak heart rate pre‐dobutamine. These were then also followed by post hoc Tukey tests for differences between individual parameters if prompted by the significance levels.

A *P* < .05 following Bonferroni correction where appropriate was considered to indicate a significant difference. Murine ECGs which demonstrated P‐wave dissociation in the analysis period were discarded for P‐wave dependent parameter analysis. All diagrams were produced with the R‐grammar of graphics package.

## References

[1] Deo R , Albert CM . Epidemiology and genetics of sudden cardiac death. Circulation. 2012;125:620‐637. 22294707 10.1161/CIRCULATIONAHA.111.023838 PMC3399522

[2] Zoni‐Berisso M , Lercari F , Carazza T , Domenicucci S . Epidemiology of atrial fibrillation: European perspective. Clin Epidemiol. 2014;6:213‐220. 24966695 10.2147/CLEP.S47385 PMC4064952

[3] Kucharska‐Newton AM , Couper DJ , Pankow JS , et al. Diabetes and the risk of sudden cardiac death, the Atherosclerosis Risk in Communities study. Acta Diabetol. 2010;47(Suppl 1):161‐168. 10.1007/s00592-009-0157-9 PMC3064263 19855920

[4] Lin PH , Lee SH , Su CP , Wei YH . Oxidative damage to mitochondrial DNA in atrial muscle of patients with atrial fibrillation. Free Radic Biol Med. 2003;35:1310‐1318. 14607530 10.1016/j.freeradbiomed.2003.07.002

[5] Bukowska A , Schild L , Keilhoff G , et al. Mitochondrial dysfunction and redox signaling in atrial tachyarrhythmia. Exp Biol Med. 2008;233:558‐574. 10.3181/0706-RM-155 18375832

[6] Kabunga P , Lau AK , Phan K , et al. Systematic review of cardiac electrical disease in Kearns‐Sayre syndrome and mitochondrial cytopathy. Int J Cardiol. 2015;181:303‐310. 25540845 10.1016/j.ijcard.2014.12.038

[7] Lin J , Handschin C , Spiegelman BM . Metabolic control through the PGC‐1 family of transcription coactivators. Cell Metab. 2005;1:361‐370. 16054085 10.1016/j.cmet.2005.05.004

[8] Huss JM , Torra IP , Staels B , Giguère V , Kelly DP . Estrogen‐related receptor alpha directs peroxisome proliferator‐activated receptor alpha signaling in the transcriptional control of energy metabolism in cardiac and skeletal muscle. Mol Cell Biol. 2004;24:9079‐9091. 15456881 10.1128/MCB.24.20.9079-9091.2004 PMC517878

[9] Arany Z , He H , Lin J , et al. Transcriptional coactivator PGC‐1 alpha controls the energy state and contractile function of cardiac muscle. Cell Metab. 2005;1:259‐271. 16054070 10.1016/j.cmet.2005.03.002

[10] Sonoda J , Mehl IR , Chong L‐W , Nofsinger RR , Evans RM . PGC‐1beta controls mitochondrial metabolism to modulate circadian activity, adaptive thermogenesis, and hepatic steatosis. Proc Natl Acad Sci U S A. 2007;104:5223‐5228. 17360356 10.1073/pnas.0611623104 PMC1829290

[11] Mootha VK , Lindgren CM , Eriksson K‐F , et al. PGC‐1α‐responsive genes involved in oxidative phosphorylation are coordinately downregulated in human diabetes. Nat Genet. 2003;34:267‐273. 12808457 10.1038/ng1180

[12] Lehman JJ , Boudina S , Banke NH , et al. The transcriptional coactivator PGC‐1alpha is essential for maximal and efficient cardiac mitochondrial fatty acid oxidation and lipid homeostasis. Am J Physiol Heart Circ Physiol. 2008;295:H185‐H196. 18487436 10.1152/ajpheart.00081.2008 PMC2494758

[13] Lai L , Leone TC , Zechner C , et al. Transcriptional coactivators PGC‐1alpha and PGC‐lbeta control overlapping programs required for perinatal maturation of the heart. Genes Dev. 2008;22:1948‐1961. 18628400 10.1101/gad.1661708 PMC2492740

[14] Lelliott CJ , Medina‐Gomez G , Petrovic N , et al. Ablation of PGC‐1β results in defective mitochondrial activity, thermogenesis, hepatic function, and cardiac performance. PLoS Biol. 2006;4:2042‐2056. 10.1371/journal.pbio.0040369 PMC1634886 17090215

[15] Gurung IS , Medina‐Gomez G , Kis A , et al. Deletion of the metabolic transcriptional coactivator PGC1beta induces cardiac arrhythmia. Cardiovasc Res. 2011;92:29‐38. 21632884 10.1093/cvr/cvr155 PMC3172981

[16] Goldbarg AN , Hellerstein HK , Bruell JH , Daroczy AF . Electrocardiogram of the normal mouse, Mus Musculus: General considerations and genetic aspects. Cardiovasc Res. 1968;2:93‐99. 5645470 10.1093/cvr/2.1.93

[17] Danik S , Cabo C , Chiello C , Kang S , Wit AL , Coromilas J . Correlation of repolarization of ventricular monophasic action potential with ECG in the murine heart. Am J Physiol Heart Circ Physiol. 2002;283:H372‐H381. 12063311 10.1152/ajpheart.01091.2001

[18] Boukens BJ , Rivaud MR , Rentschler S , Coronel R . Misinterpretation of the mouse ECG: “musing the waves of Mus musculus”. J Physiol. 2014;21:4613‐4626. 10.1113/jphysiol.2014.279380 PMC4253466 25260630

[19] Jeevaratnam K , Zhang Y , Guzadhur L , et al. Differences in sino‐atrial and atrio‐ventricular function with age and sex attributable to the Scn5a+/− mutation in a murine cardiac model. Acta Physiol (Oxf). 2010;200:23‐33. 20331542 10.1111/j.1748-1716.2010.02110.x

[20] Martin CA , Zhang Y , Grace AA , Huang CL‐H . In vivo studies of Scn5a+/− mice modeling Brugada syndrome demonstrate both conduction and repolarization abnormalities. J Electrocardiol. 2010;43:433‐439. 20638671 10.1016/j.jelectrocard.2010.05.015 PMC3712183

[21] Wu J , Zhang Y , Zhang X , et al. Altered sinoatrial node function and intra‐atrial conduction in murine gain‐of‐function Scn5a+/KPQ hearts suggest an overlap syndrome. Am J Physiol Heart Circ Physiol. 2012;302:H1510‐H1523. 22287583 10.1152/ajpheart.00357.2011 PMC3330789

[22] Zhang Y , Fraser JA , Jeevaratnam K , et al. Acute atrial arrhythmogenicity and altered Ca(2 + ) homeostasis in murine RyR2‐P2328S hearts. Cardiovasc Res. 2011;89:794‐804. 20621925 10.1093/cvr/cvq229 PMC3039245

[23] Brubaker PH , Kitzman DW . Chronotropic incompetence: Causes, consequences, and management. Circulation. 2011;123:1010‐1020. 21382903 10.1161/CIRCULATIONAHA.110.940577 PMC3065291

[24] Massumi RA , Ali N . Accelerated isorhythmic ventricular rhythms. Am J Cardiol 1970;26:170‐185. 5455536 10.1016/0002-9149(70)90777-0

[25] Liu M , Liu H , Dudley SC . Reactive oxygen species originating from mitochondria regulate the cardiac sodium channel. Circ Res. 2010;107:967‐974. 20724705 10.1161/CIRCRESAHA.110.220673 PMC2955818

[26] Wang J , Wang H , Zhang Y , Gao H , Nattel S , Wang Z . Impairment of HERG K+ Channel Function by Tumor Necrosis Factor‐α: Role of reactive oxygen species as a mediator. J Biol Chem. 2004;279:13289‐13292. 14973143 10.1074/jbc.C400025200

[27] Terentyev D , Gyorke I , Belevych AE , et al. Redox modification of ryanodine receptors contributes to sarcoplasmic reticulum Ca(2 + ) leak in chronic heart failure. Circ Res. 2008;103:1466‐1472. 19008475 10.1161/CIRCRESAHA.108.184457 PMC3274754

[28] Bovo E , Lipsius SL , Zima AV . Reactive oxygen species contribute to the development of arrhythmogenic Ca^2+^ waves during β‐adrenergic receptor stimulation in rabbit cardiomyocytes. J Physiol. 2012;590:3291‐3304. 22586224 10.1113/jphysiol.2012.230748 PMC3459043

[29] Brown DA , O'Rourke B . Cardiac mitochondria and arrhythmias. Cardiovasc Res. 2010;88:241‐249. 20621924 10.1093/cvr/cvq231 PMC2980943

[30] Akar FG , O'Rourke B . Mitochondria are sources of metabolic sink and arrhythmias. Pharmacol Ther. 2011;131:287‐294. 21513732 10.1016/j.pharmthera.2011.04.005 PMC3138548

[31] Smyth JW , Hong TT , Gao D , et al. Limited forward trafficking of connexin 43 reduces cell‐cell coupling in stressed human and mouse myocardium. J Clin Invest. 2010;120:266‐279. 20038810 10.1172/JCI39740 PMC2798685

[32] Jeevaratnam K , Rewbury R , Zhang Y , et al. Frequency distribution analysis of activation times and regional fibrosis in murine Scn5a+/− hearts: The effects of ageing and sex. Mech Ageing Dev. 2012;133:591‐599. 22968175 10.1016/j.mad.2012.07.006 PMC3466423

[33] Jeevaratnam K , Guzadhur L , Goh Y , Grace A , Huang CL‐H . Sodium channel haploinsufficiency and structural change in ventricular arrhythmogenesis. Acta Physiol. 2016;216:186‐202. 10.1111/apha.12577 26284956

[34] Gazoti Debessa CR , Mesiano Maifrino LB , Rodrigues de Souza R . Age related changes of the collagen network of the human heart. Mech Ageing Dev. 2001;122:1049‐1058. 11389923 10.1016/s0047-6374(01)00238-x

[35] Xie Y , Garfinkel A , Camelliti P , Kohl P , Weiss JN , Qu Z . Effects of fibroblast‐myocyte coupling on cardiac conduction and vulnerability to reentry: A computational study. Heart Rhythm. 2009;6:1641‐1649. 19879544 10.1016/j.hrthm.2009.08.003 PMC3013501

[36] Chilton L , Giles WR , Smith GL . Evidence of intercellular coupling between co‐cultured adult rabbit ventricular myocytes and myofibroblasts. J Physiol. 2007;583:225‐236. 17569734 10.1113/jphysiol.2007.135038 PMC2277230

[37] Jones SA , Lancaster MK , Boyett MR . Ageing‐related changes of connexins and conduction within the sinoatrial node. J Physiol. 2004;560:429‐437. 15308686 10.1113/jphysiol.2004.072108 PMC1665255

[38] Nisbet AM , Camelliti P , Walker NL , et al. Prolongation of atrio‐ventricular node conduction in a rabbit model of ischaemic cardiomyopathy: Role of fibrosis and connexin remodelling. J Mol Cell Cardiol. 2016;94:54‐64. 27021518 10.1016/j.yjmcc.2016.03.011 PMC4873602

[39] Sullivan DE , Ferris M , Pociask D , Brody AR . The latent form of TGFβ 1 is induced by TNFα through an ERK specific pathway and is activated by asbestos‐derived reactive oxygen species in vitro and in vivo. J Immunotoxicol. 2008;5:145‐149. 18569384 10.1080/15476910802085822

[40] Davies L , Jin J , Shen W , et al. Mkk4 is a negative regulator of the transforming growth factor beta 1 signaling associated with atrial remodeling and arrhythmogenesis with age. J Am Heart Assoc. 2014;3:1‐19. 10.1161/JAHA.113.000340 PMC4187508 24721794

[41] Dai D‐F , Santana LF , Vermulst M , et al. Overexpression of catalase targeted to mitochondria attenuates murine cardiac aging. Circulation. 2009;119:2789‐2797. 19451351 10.1161/CIRCULATIONAHA.108.822403 PMC2858759

[42] Sovari AA , Rutledge CA , Jeong E‐M , et al. Mitochondria oxidative stress, connexin‐43 remodeling, and sudden arrhythmic death. Circ Arrhythm Electrophysiol. 2013;6:623‐631. 23559673 10.1161/CIRCEP.112.976787 PMC3716298

[43] Bhuiyan ZA , Van Den Berg MP , Van Tintelen JP , et al. Expanding spectrum of human RYR2‐related disease: New electrocardiographic, structural, and genetic features. Circulation. 2007;116:1569‐1576. 17875969 10.1161/CIRCULATIONAHA.107.711606

[44] Zhang Y , Wu J , King JH , Huang CL‐H , Fraser JA . Measurement and interpretation of electrocardiographic QT intervals in murine hearts. Am J Physiol Heart Circ Physiol. 2014;306:H1553‐H1557. 24705556 10.1152/ajpheart.00459.2013 PMC4042200

[45] Jeevaratnam K , Poh Tee S , Zhang Y , et al. Delayed conduction and its implications in murine Scn5a+/− hearts: Independent and interacting effects of genotype, age, and sex. Pflugers Arch. 2011;461:29‐44. 21127902 10.1007/s00424-010-0906-1 PMC3016216

[46] King JH , Wickramarachchi C , Kua K , et al. Loss of Nav1.5 expression and function in murine atria containing the RyR2‐P2328S gain‐of‐function mutation. Cardiovasc Res. 2013;99:751‐759. 23723061 10.1093/cvr/cvt141

[47] Ning F , Luo L , Ahmad S , et al. The RyR2‐P2328S mutation downregulates Nav1.5 producing arrhythmic substrate in murine ventricles. Pflügers Arch Eur J Physiol. 2016;468:655‐665. 26545784 10.1007/s00424-015-1750-0 PMC4792352

[48] Gollob MH , Redpath CJ , Roberts JD . The short QT syndrome: Proposed diagnostic criteria. J Am Coll Cardiol. 2011;57:802‐812. 21310316 10.1016/j.jacc.2010.09.048

[49] Antzelevitch C , Pollevick GD , Cordeiro JM , et al. Loss‐of‐function mutations in the cardiac calcium channel underlie a new clinical entity characterized by ST‐segment elevation, short QT intervals, and sudden cardiac death. Circulation. 2007;115:442‐449. 17224476 10.1161/CIRCULATIONAHA.106.668392 PMC1952683

[50] Billman GE . The cardiac sarcolemmal ATP‐sensitive potassium channel as a novel target for anti‐arrhythmic therapy. Pharmacol Ther. 2008;120:54‐70. 18708091 10.1016/j.pharmthera.2008.07.004

[51] Yan X , Ma J , Zhang P . Modulation of KATP currents in rat ventricular myocytes by hypoxia and a redox reaction. Acta Pharmacol Sin. 2009;30:1399‐1414. 19801996 10.1038/aps.2009.134 PMC4007324

[52] Mitchell GF , Jeron A , Koren G . Measurement of heart rate and Q‐T interval in the conscious mouse. Am J Physiol. 1998;274:H747‐H751. 9530184 10.1152/ajpheart.1998.274.3.H747

